# Naso-Orbito-Ethmoid Fractures: Refining the Role of Wires and Plates

**DOI:** 10.3390/cmtr18040053

**Published:** 2025-12-18

**Authors:** Preston Leader, Kelsey Karnik, Anthony Mangino, Clayton Bobo, Thomas Gal

**Affiliations:** 1 Department of Otolaryngology-Head and Neck Surgery, University of Kentucky, Lexington, KY 40536, USA; 2 Department of Biostatistics, University of Kentucky, Lexington, KY 40536, USA; 3 School of Medicine, University of Kentucky, Lexington, KY 40536, USA; clayton.bobo@uky.edu

**Keywords:** naso-orbito-ethmoid fractures, intercanthal wiring, telecanthus

## Abstract

Background: Naso-orbital-ethmoid (NOE) fractures represent complex midface injuries that challenge aesthetic and functional reconstruction. This study evaluates the efficacy of techniques restoring intercanthal distance following operative repair of NOE fractures. Methods: A retrospective case series was conducted of adults undergoing NOE fracture repair between 2010 and 2022. CPT codes were used to identify patients, with inclusion based on radiographic confirmation of NOE fractures. Demographic data, fracture classification, operative techniques, and pre- and post-operative CT measurements of intercanthal distance were analyzed by fracture type and type of repair. Results: 191 patients were identified, mostly male (80%), with Type I fractures being most common (66%). Intercanthal wiring was used in 14% of cases, most frequently for Type II and III fractures. Of the 100 patients with post-operative comparison imaging, the median intercanthal distance improved from 34 mm to 31 mm. Intercanthal wiring yielded greater median distance correction. All patients achieved restoration of intercanthal distance within normal limits regardless of repair technique. Conclusions: Operative repair of NOE fractures using either plating or intercanthal wiring effectively restores normal intercanthal distance. While intercanthal wiring remains valuable in severe fractures, it may not be universally necessary. Further study is needed to refine the role of these repair techniques.

## 1. Introduction

Naso-orbital ethmoid (NOE) complex fractures are complicated midface fractures that are functionally and aesthetically challenging to repair. The main component of the NOE fracture is the displacement of the medial wall of the orbit with associated medial canthal tendon (MCT) attachment. Markowitz’s 1991 classification system was universally used to grade NOE fractures based on the MCT position and associated central bone fragment [1]. Many techniques have been utilized throughout history to reduce NOE fractures. The majority of the primary literature outlining these techniques was published before the adoption of the Markowitz NOE classification system [1,2].

The MCT supports the medial canthus, positions the eyelid and globe, and plays a role in the functioning and support of the lacrimal system. The disruption of the NOE confluence presents clinically with varying degrees of telecanthus. Repairing the different degrees of NOE fractures centers around properly positioning the MCT and restoring the intercanthal distance through the use of miniplates and intercanthal wiring. The frequency of fracture and repair difficulty is inversely related to the NOE type. Intercanthal wiring can be difficult to place effectively. Different techniques to place intercanthal wiring have been described in the literature [1,3,4,5].

Using a multidisciplinary institutional experience, the objective of this study was to review the experience with naso-orbital-ethmoid fractures. This represents the experience of multiple surgeons across multiple specialties. It is an observational study and is not meant as a commentary on the techniques that were used. Rather, it is an attempt to refine the role, if any, of wiring in the outcome of fracture reduction. The goal was a quantitative assessment of the use of plating and intercanthal wiring and their association with the restoration of intercanthal distance.

## 2. Materials and Methods

A review of this retrospective case series was approved by the University of Kentucky Institutional Review Board (IRB # 78257). Data was compiled through the Center of Clinical and Translational Science and extracted from the electronic medical records (EMRs) of patients in the University of Kentucky Healthcare system. The study period was from 1 January 2010 to 18 May 2022. To identify patients who underwent operative repair of NOE fractures, CPT codes 21338, 21339, 21342, 21344,21346, 21347, 21348, 21432, 21433, 21435, and 21436 were used. Coding was selected to capture isolated naso-orbital-ethmoid fractures, as well as frontal sinus fractures and LeFort II and III fractures, where the NOE complex may be involved but not specifically coded.

Variables of interest collected included demographic information, mechanism of injury, naso-orbital ethmoid fracture type, concomitant facial fractures, intercanthal distance pre- and post-operative on CT imaging, surgical technique employed, use of intercanthal wiring, and use of plates for fixation. Patients 18 years and older who underwent operative repair during the specified study period were included in the study.

Pre-operative CT scans and radiology reports were reviewed to confirm NOE fractures. NOE fracture was defined as a fracture involving the medial orbit/lateral nasal wall extending through the nasolacrimal bone [6]. Associated fractures were identified and included in the analysis. Thus, patients classified as having a unilateral NOE frequently had contralateral LeFort injuries, despite the contralateral nasal segment not meeting criteria for classification as an NOE. Patients with no identifiable radiographic evidence of NOE fracture were excluded.

**Figure 1:**
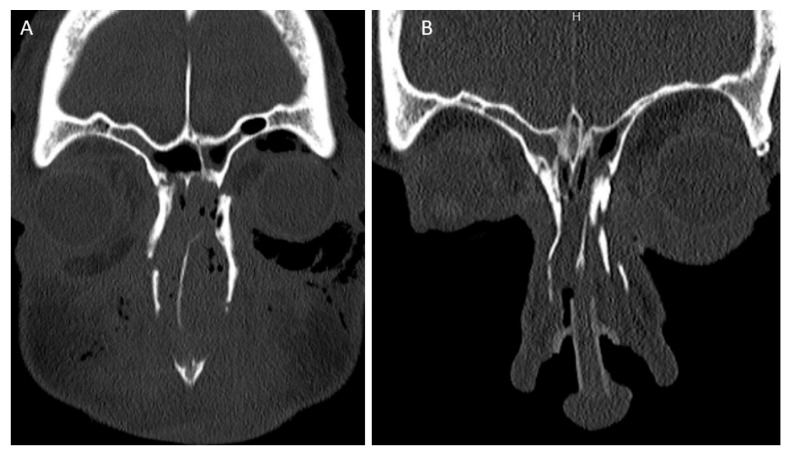
Pre-(**A**) and post-(**B**) operative reduction in canthal fragment (fixation not completely shown).

Both intercanthal and interpupillary distances were measured on pre-operative scans and available post-operative CT scans. Representative scans are shown in Figure 1. These measurements were compared to normative values for intercanthal and interpupillary distance obtained from the literature for the population [7,8].

Intercanthal Distances:

- Men: 31.9 ± 2.2 mm (29.7, 34.1)
- Women: 30.7 ± 2.6 mm (28.1, 33.1)

Interpupillary Distances:

- Men: 64.0 ± 3.45 mm (60.55, 67.45)
- Women: 61.7 ± 3.65 mm (58.05, 65.35)

**Figure 2:**
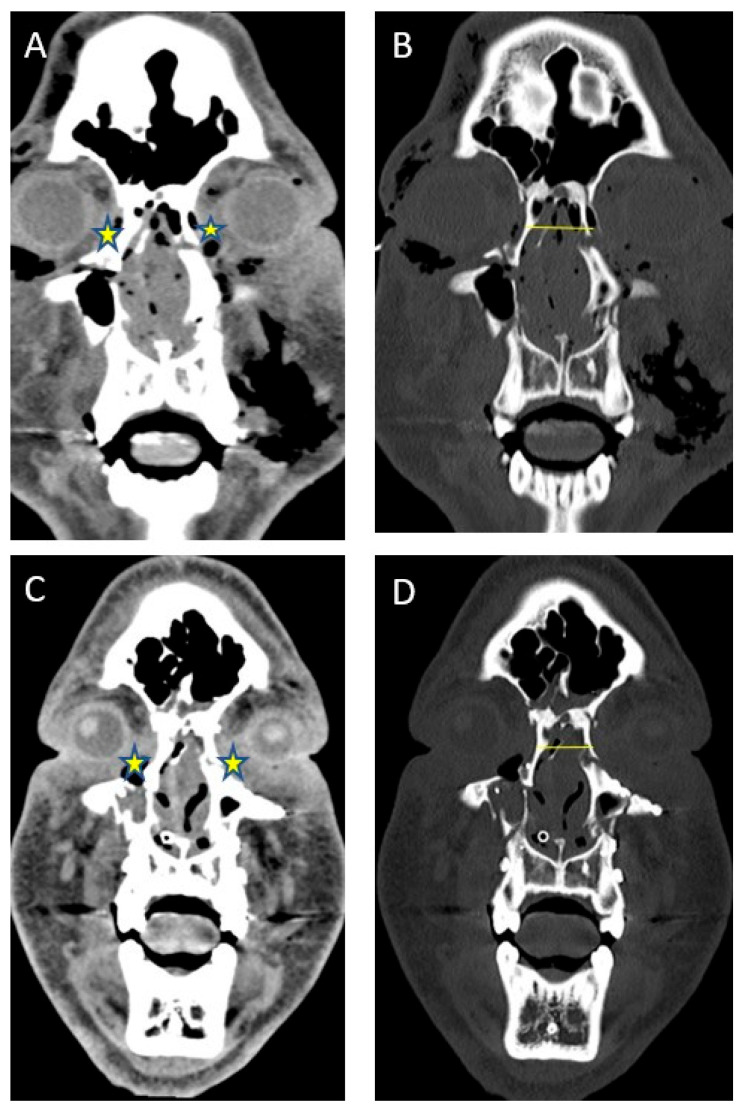
Intercanthal distance measurement. (**A**) Pre-operative soft tissue imaging showing canthal tendons (stars). (**B**) Pre-operative bone windows showing measurement of intercanthal distance (line). (**C**) Post-operative soft tissue imaging of canthal tendons (stars). (**D**) Post-operative bone windows showing measurement of intercanthal distance (line).

When available, intercanthal and interpupillary distances were measured on pre- and post-operative CT scans. All scans were reviewed and measured by the authors. When possible, the canthal fragment(s) were identified on pre-operative scans using both soft tissue and bone windows. The intercanthal distance was then measured on the scan. The measurements were then compared to the post-operative result. It was frequently necessary to identify the reduced canthal fragment on post-operative scans and then find it on the pre-operative scans for measurement, as this is not always readily identified. While this technically represents interfragmentary distance, the distance of the fragment was measured at the perceived site of the canthal tendon. Measurement error, if present, occurred in a consistent manner, producing reliable results. Representative images demonstrating this approach are shown in Figure 2.

Values were defined as “in range” if the measured value was within the normative data above, or “out of range” if the value was greater than the normative data. Findings were stratified by both types of fracture and the use of wiring for both unilateral and bilateral NOE fractures. Given the duplication of findings with no significant differences in outcome, only intercanthal distance data is presented. Identified patients without available CT scans or operative reports, as well as patients who underwent closed reduction, were excluded.

Statistical tests conducted were two-sided, with statistical significance defined as a *p*-value ≤ 0.05. All analyses were performed in the R programming language, version 4.3.1 (R Foundation for Statistical Computing, Vienna, Austria). Wilcoxon rank sum, Fisher’s exact, or Pearson’s Chi-squared tests were used depending on the format of the variable. Quantitative variables utilize the Wilcoxon rank sum test. Categorical variables are tested either with the Chi-squared or Fisher’s exact tests. When cell counts fall below 5, Fisher’s exact tests were used to quantify statistical significance.

## 3. Results

One hundred ninety-one patients were identified by CPT code. 52 patients were excluded (closed reduction, no preop CT imaging, unavailable operative report), with 139 patients were included in the final cohort.

The mean age was 41.7 ± 17.3. 98% (*n* = 137) of the patients were white, and 80% (*n* = 111) were male. The most common mechanism of injury was motor vehicle collision (43% *n* = 60). A total of 57% (*n* = 80) of patients had unilateral NOE fractures. Overall, 91% (*n* = 127) of patients had clinically evident NOE fractures as documented on physical exam (Table 1). The most common fracture type was type 1 NOE fracture (66% *n* = 92). Type 3 NOE fractures accounted for 5% (*n* = 7). Finally, 56% (*n* = 79) of patients had an associated LeFort fracture. A summary of demographic information is presented in Table 1.

Nineteen (20.9%) of these patients underwent intercanthal wiring. Of the 19 patients, 11 (57%) had type II fractures, 5 (26%) type III, and 3 (15%) type I. In the 120 patients with plate-only repair, the majority of fractures were Type I (*N* = 89, 74%). For clarity, these data are presented in Table 2.

Overall, 72% (*n* = 100) of patients had postoperative films to review for comparative analysis. Median radiographic pre-operative intercanthal distance was 34 mm (Interquartile range 32–37 mm). Post-operative median radiographic intercanthal distance was 31 mm (interquartile range 30–32 mm). Pre-operatively, 26 patients (28.6%) were classified as “out of range” when compared to normative values. All patients were classified as “in range” post-operatively, regardless of operative technique.

**Figure 3:**
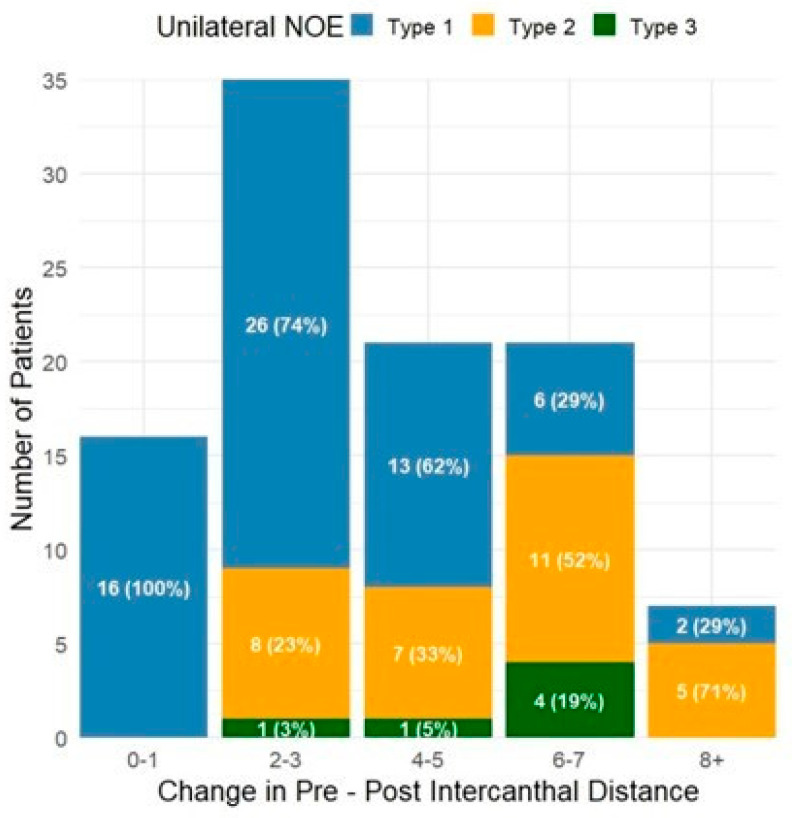
Intercanthal distance change (mm) for unilateral fractures by NOE Type. Change in pre- and post-operative intercanthal distance stratified by fracture type, with the total number of each fracture and percentage.

**Figure 4:**
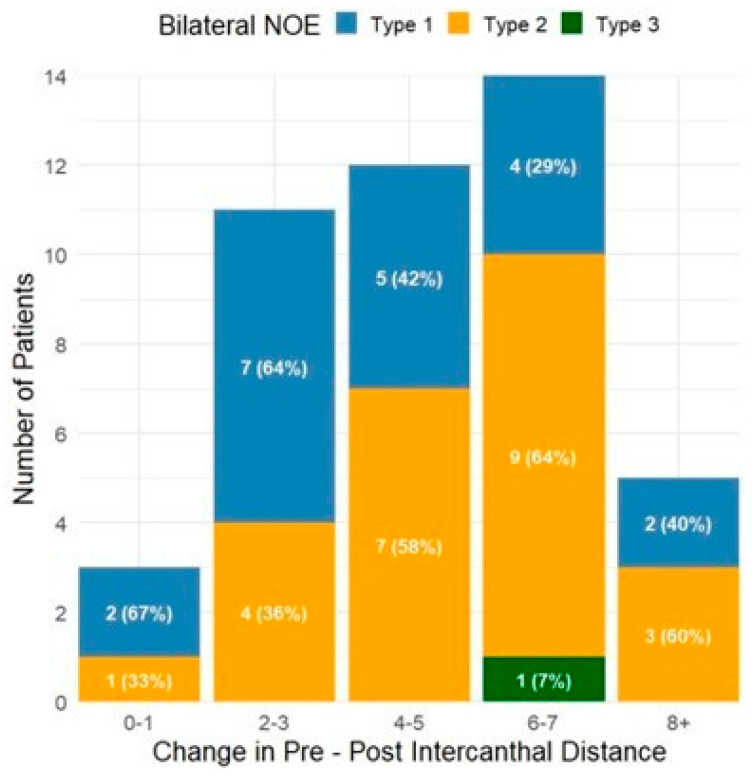
Intercanthal distance change (mm) for bilateral fractures by NOE Types. Change in pre- and post-operative intercanthal distance stratified by fracture type, with the total number of each fracture and percentage.

**Figure 5:**
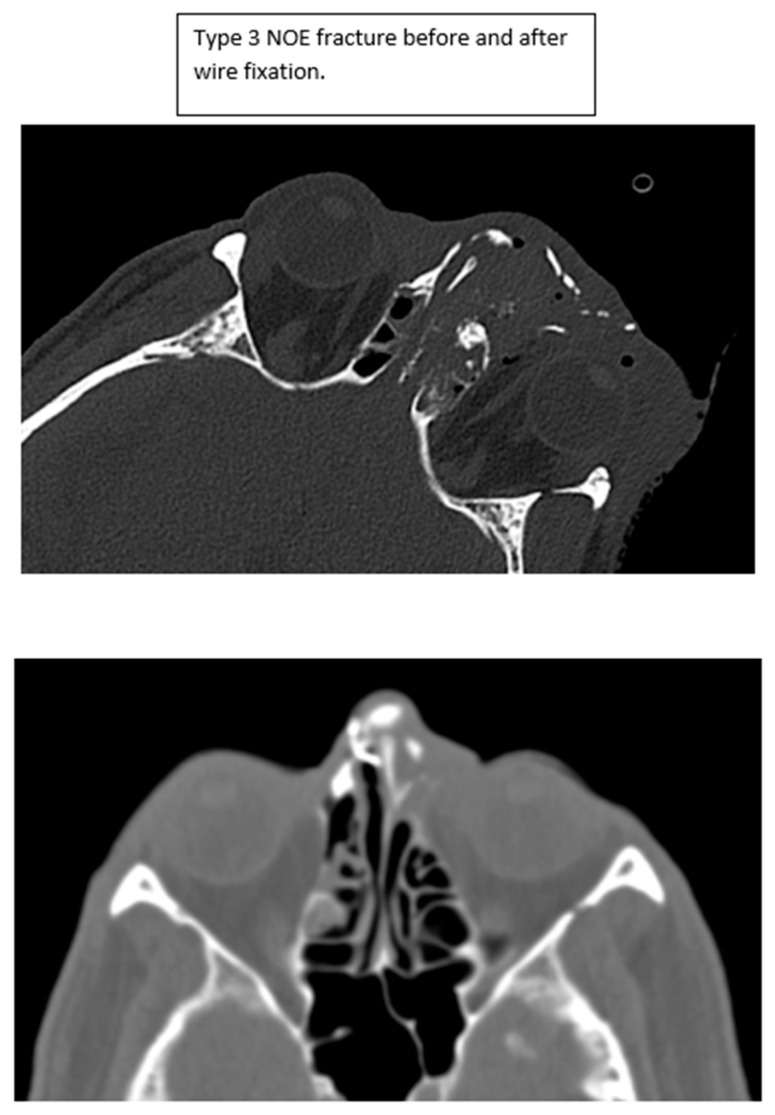
Type 3 NOE fracture CT imaging before and after intercanthal wiring.

**Figure 6:**
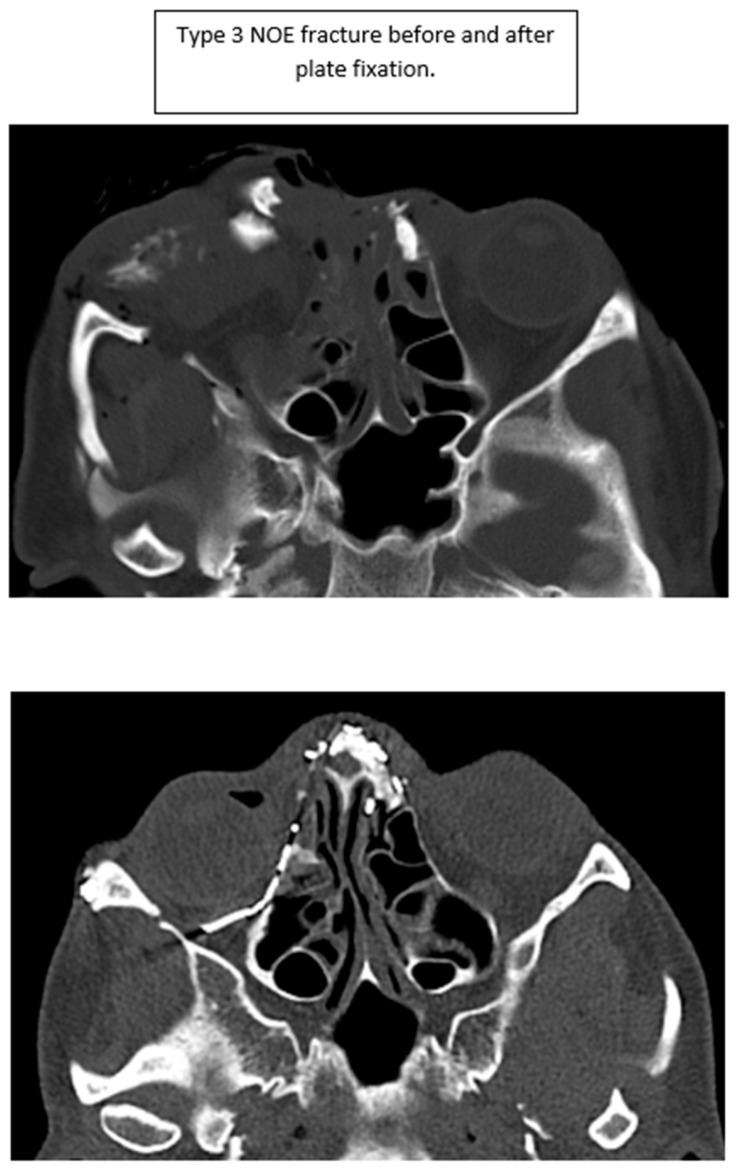
Type 3 NOE fracture CT imaging before and after plate fixation.

Millimeters change from pre-operative to post-operative intercanthal distance for both unilateral and bilateral NOE fractures are presented in Figure 3 and Figure 4, respectively. For unilateral fractures, the majority (*N* = 30, 43%) were Type I fractures with a change in intercanthal distance of 2–3 mm. Bilateral fractures were less common with a wider distribution, with the most common observed to be Type II/III fractures with a change in intercanthal distance of 6–7 mm (*N* = 8, 38%). Change in intercanthal distance comparing patients treated with intercanthal wiring versus plate-only repair is shown in Figure 5 and Figure 6. The majority of patients were treated with plate-only repair with an observed change of 2–3 mm. What is most significant, however, is the group with an intercanthal distance change of >6 mm. Ten patients were treated with intercanthal wiring, compared to 18 with plate-only repair. Only five patients in the intercanthal wiring group had type III fractures, all unilateral, four of which had an observed intercanthal distance change of >6 mm. All patients were reduced to within normal range post-operatively. The implication is that severe telecanthus is not an absolute indication for intercanthal wiring.

## 4. Discussion

NOE fractures are technically challenging fractures to repair. The complex anatomy and access to the midface add to the challenge. To our knowledge, this is the first quantitative study of the efficacy of repairing NOE fractures.

As previously outlined in our systematic review, the treatment of NOE fractures transitioned from closed reduction with external plate fixation techniques to improved results with an open reduction approach using interfragmental wiring fixation in the 1960s [1,2,9,10,11]. Stranc et al. in the 1970s advocated for transnasal wiring of the MCT and exploration through existing lacerations [12]. Miniplates with screw fixation became available in the mid-1980s, with contoured miniplates providing added frontal maxillary stability [13]. Markowitz’s work transitioned from interfragmentary wiring to microplates with screw fixation, ultimately creating the modern classification system in 1991 with a specific repair technique [1,9]. Open reduction internal fixation with microplates and screws was one of the most important contributions and is considered the gold standard today for repair of NOE fractures.

Type I NOE fractures represent a noncomminuted fracture with an attached medial canthal tendon to a large bony segment and represent the most common fracture type [1]. Management can include observation or operative repair, depending on fragment displacement and mobility. Our study focused on operative repair and excluded those patients who were observed.

Type II and Type III NOE fractures represent more severely comminuted fractures with and without an attached medial canthal tendon, respectively [9]. Type II and type III fractures present an added level of complexity. Proper fixation of the canthal-bearing bone fragment is the most critical component of NOE fracture repair. As previously reviewed, MCT is rarely avulsed from blunt trauma and is often iatrogenic if encountered [1]. The need for transnasal wiring to restore intercanthal distance has historically been determined by the canthal bearing bone fragment and the degree of comminution. The vector for transnasal wiring needs to be posterior and superiorly oriented to the lacrimal fossa to prevent horizontal displacement. Transnasal wiring, although challenging to place, remains the current standard for MCT avulsion. Primary repair of telecanthus is essential to provide the best aesthetic and functional results [14].

The issue raised by the results of this study is whether intercanthal wiring is necessary for the management of even the most severe NOE fractures. Without question, intercanthal wiring is the most challenging technique to perform properly in the management of maxillofacial trauma. First and foremost, it is essential when identifying the medial canthal tendon to identify the soft tissue structure first, so that whatever bony attachment that remains can be handled. The structure is very adherent to the bone and is infrequently completely avulsed as a result of the injury. Stripping all of the soft tissue from the bone in this region may result in a situation that is difficult to recreate. Thus, there is a high risk of iatrogenic conversion of a type II fracture to a type III fracture.

Management of NOE fractures is the one situation in craniomaxillofacial trauma where both soft tissue and bone repair are paramount. However, one may facilitate the other. For type I and larger segment type II fractures, accurate reduction in the bone will allow the soft tissue to follow. For smaller segment type II and certainly type III fractures, this becomes more challenging. However, an accurate reduction in the bone will facilitate the placement of the soft tissue. Intercanthal wiring remains an effective means for proper soft tissue repair and positioning of the medial canthal tendon(s) with restoration of the intercanthal distance in experienced hands. Alternatively, the tendon and/or its attached fragment can be effectively positioned by securing it to the ipsilateral bone or hardware. Using either a suture through the tendon itself or a drill hole through the attached segment that might not otherwise be secured with plating, the tendon segment can be resuspended accurately without the added difficulty and inherent inaccuracy of reaching the other side. This can be performed on both sides of the bilateral fracture without having to try to secure one to the other. Using a “Y” or “T” bent plate can allow for a reduction in the bone segment, while using the “branch” to secure the tendon more superiorly and posteriorly with greater precision than one might be able to achieve with crossing wires. Intercanthal wiring was observed to provide the largest median intercanthal correction (6 mm). However, the largest intercanthal correction using plates alone was 10 mm, so this may be a function of sample size.

One of the biggest limitations of this retrospective review is that we are unable to accurately determine the true indications for intracanthal wiring at the time of surgery. Greater difficulty with surgical repair may be encountered, but not reflected in retrospective fracture staging. This cohort represents the work of multiple services and multiple surgeons from otolaryngology, plastic surgery, and oral maxillofacial surgery. The prevalence of the use of intercanthal wiring may simply represent surgeon preference. Additionally, there is some confusion regarding the utility of intercanthal wiring in the setting of unilateral NOE fractures. It is important to recognize that a significant number of patients classified as unilateral NOE fractures occurred in the setting of bilateral LeFort fractures. A strict classification criterion was used to classify patients as having an NOE fracture in this cohort. It was difficult to define the status of the contralateral segment in such a way as to determine whether wiring should or should not be used. Although this explains the frequency of unilateral fractures, it does not in any way allow for the explanation or justification of the use of wiring.

It is important to make the distinction between intercanthal distance and telecanthus. Telecanthus is a clinical diagnosis made on physical examination. This is a retrospective radiographic study. While it is without question that clinical pre- and post-operative measurements would have been valuable, the scarcity of these data in the medical records limited the ability to make any associations or conclusions. It is certainly conceivable that a widened intercanthal distance was present in the absence of clinical telecanthus, and vice versa. Corresponding clinic images would be unquestionably useful. The clinical implication of this radiographic study is certainly one of its limitations.

What is most important is that, regardless of technique, appropriate management of NOE fractures results in effective restoration of intercanthal distance regardless of fracture severity. While it is possible that less successful outcomes were simply not captured with post-operative imaging, it would stand to reason that less-than-optimal repair, assuming follow-up, would be more likely to be captured by post-operative imaging. The true optimal approach to NOE fractures undoubtedly requires further study. It may remain a function of both surgeon preference and the variability of the injury.

## 5. Conclusions

Naso-orbital-ethmoid fractures remain a functional and aesthetic challenge. Regardless of approach, restoration of normal intercanthal distance is achievable. True indications and alternatives to intracanthal wiring in the setting of advanced fractures require further study.

## Figures and Tables

**Table 1:** Patient demographics.

Variable	Overall, *N* = 139
Age [Mean (SD)]	41.70 (17.26)
Race	
White	137 (98.56%)
Other	2 (1.44%)
Ethnicity	
Not Hispanic	133 (95.683%)
Hispanic	6 (4.3165%)
Gender	
Female	28 (20.14%)
Male	111 (79.86%)
Injury Mechanism	
MVA	83 (59.71%)
Assault	21 (15.11%)
Other Blunt Trauma	28 (20.14%)
GSW	7 (5.04%)
NOE type (radiographic)	
Unilateral	80 (57.55%)
Bilateral	59 (42.45%)
NOE Classification	
Unilateral	
Type I	55 (39.57%)
Type II	22 (15.83%)
Type III	6 (4.32%)
Bilateral	
Type I	40 (28.78%)
Type II	18 (12.95%)
Type III	1 (0.72%)
Total	
Type I	92 (66.19%)
Type II	40 (28.78%)
Type III	7 (5.04%)
Frontal Sinus Fracture	
Yes	69 (49.64%)
No	67 (48.20%)
NA	3 (2.16%)
Le Fort Fracture	
Yes	79 (56.83%)
No	60 (43.17%)
Mandible Fracture	
Yes	28 (20.14%)
No	108 (77.70%)
NA	3 (2.16%)
Intercanthal Wiring	
Yes	19 (13.67%)
No	120 (86.33%)
Pre-operative intercanthal distance (imaging)	
Median (IQR)	34 (32, 37)
Post-operative intercanthal distance (imaging)	
Median (IQR)	31 (30, 32)

**Table 2:** Intercanthal wiring vs. plating by fracture type with total number and percentages. Chi-square test performed with *p* < 0.001.

NOE Classification Fracture Type	Wiring + Plate *n* = 19	Plates Only *n* = 120	
Type I	3 (15.79%)	89 (74.17%)	* *p* < 0.001
Type II	11 (57.90%)	29 (24.17%)	
Type III	5 (26.32%)	2 (1.67%)	

* Chi-squared test *p*-value.

## Data Availability

Data is unavailable due to privacy and ethical restrictions.

## Abbreviations

The following abbreviations are used in this manuscript:

NOE	Naso-Orbito-Ethmoid
MCT	Medial Canthal Tendon
CT	Computerized Tomography
